# Interstitial Lung Disease Associated With Autoimmune Rheumatic Diseases: Checklists for Clinical Practice

**DOI:** 10.3389/fmed.2021.732761

**Published:** 2021-10-15

**Authors:** Silvia Laura Bosello, Lorenzo Beretta, Nicoletta Del Papa, Sergio Harari, Stefano Palmucci, Alberto Pesci, Gilda Rechichi, Francesco Varone, Marco Sebastiani

**Affiliations:** ^1^Rheumatology Unit, A. Gemelli University Hospital Foundation - Istituto di Ricerca e Cura a Carattere Scientifico (IRCCS), Catholic University of the Sacred Heart, Rome, Italy; ^2^Scleroderma Unit, Istituto di Ricerca e Cura a Carattere Scientifico (IRCCS) Ca' Granda Foundation Ospedale Maggiore Policlinico di Milan, Milan, Italy; ^3^Rheumatology Department, Scleroderma Clinic, ASST Pini-CTO, Milan, Italy; ^4^Department of Medical Sciences, San Giuseppe Hospital MultiMedica Istituto di Ricerca e Cura a Carattere Scientifico (IRCCS), Milan, Italy; ^5^Department of Clinical Sciences and Community Health, Università Degli Studi di Milano, Milan, Italy; ^6^Radiology Unit I, Department of Medical Surgical Sciences and Advanced Technologies GF Ingrassia, University Hospital Policlinico “G. Rodolico-San Marco”, University of Catania, Catania, Italy; ^7^Pneumological Clinic of the University of Milan-Bicocca, ASST-Monza, Monza, Italy; ^8^Radiology Unit, University Hospital San Gerardo, ASST-Monza, Monza, Italy; ^9^Pneumology Unit, A. Gemelli University Hospital Foundation Istituto di Ricerca e Cura a Carattere Scientifico (IRCCS), Rome, Italy; ^10^Rheumatology Unit, University of Modena and Reggio Emilia, Italy

**Keywords:** interstitial lung disease, autoimmune rheumatic diseases, multidisciplinary team, nominal group technique, Delphi panel survey, red flags and referral indications, consensus, ARD-ILD

## Abstract

**Background:** Interstitial lung diseases (ILDs) are often associated with rheumatic diseases. Their early diagnosis and management are not only difficult, but also crucial, because they are associated with major morbidity and mortality and can be the first cause of death in autoimmune rheumatic diseases (ARDs).

**Objectives:** By using methodologies, such as Nominal Group Technique (NGT) and Delphi Survey, the aims of this study were (1) to measure consensus between pulmonologists, radiologists, and rheumatologists experienced in the management of ARD-ILD; (2) to highlight the importance of a multidisciplinary approach; and (3) to provide clinicians with a practical tool aimed at improving the prompt recognition and follow-up of ILD associated with ARDs and of any possible rheumatic conditions underlying ILD.

**Results:** During the NGT round, the Steering Committee defined 57 statements to be used in the Delphi survey. A total of 78 experts participated in the Delphi survey, namely 28 pulmonologists, 33 rheumatologists, and 17 radiologists. During this round, consensus on agreement was reached in 47 statements, while disagreement was not reached in any statements. A secondary questionnaire was drafted by the Steering Committee to obtain clearer indications on ILD-ARD “red-flags” and follow-up. Delphi Panelists took part also in the second-questionnaire survey. Answers from both surveys were used to draft two checklists of “red flags” sign or symptom suggestive of ILD and ARD, respectively, and two checklists on identification and monitoring of rheumatoid arthritis (RA) and systemic sclerosis (SSc) ILD.

**Limitations:** This study is a consensus work, which cannot produce empiric data, and is limited to the Italian scenario.

**Conclusions:** This work showed a high level of agreement, but also shows some divergent opinions between different experts. This underlines the importance of a multidisciplinary approach. Eventually, we believe the drafted checklists can help clinicians in the diagnosis and follow-up of ILD-ARD.

## Introduction

Interstitial lung diseases (ILDs) encompass a heterogeneous group of clinical conditions characterized by fibrosis of and/or inflammation the lungs (1). A common cause of ILD is represented by rheumatic diseases; in these conditions, lung involvement is not only common, but can be the main organ involvement (2, 3). Systemic sclerosis (SSc), rheumatoid arthritis (RA), antisynthetase syndrome, Sjogren's syndrome, mixed connective tissue disease (CTD), idiopathic inflammatory myopathies, and systemic lupus erythematosus are often associated with ILD (4). Moreover, a recent international consensus statement proposed “interstitial pneumonia with autoimmune features” as a new definition for ILD underlined by systemic autoimmune condition and not classifiable as any definite CTD, emphasizing the relationship between ILD and autoimmune response (5, 6). In patients with rheumatic diseases, ILD is difficult to diagnose at an early stage, and can be associated with major morbidity and mortality, or even be the leading cause of death (7–13).

Interstitial lung diseases should be managed, from its diagnosis, by a multidisciplinary team (MDT) composed of at least one pulmonologist, one radiologist, and one pathologist (2–4, 14). However, since phenotypic features of both ILDs and systemic autoimmune disorders often overlap, the patient's assessment should not be limited to clinical, radiological, and pathological evaluation, but should also include a clinical–immunological evaluation. The inclusion of an expert rheumatologist to the MDT can significantly reduce invasive procedures and increase diagnostic accuracy (1, 3, 4, 15). Nonetheless, in daily practice, it could be of use having specific and easy-to-use recommendations to improve the diagnosis and follow-up even for clinicians without specific experience in ARD-ILD or when an MDT is not available. This would help reduce diagnostic timing, which is of outmost importance, since a prompt recognition of the pathology would result in better outcomes.

Aims of this work were (1) to measure consensus between pulmonologists, radiologists, and rheumatologists experienced in the management of ARD-ILD; (2) to highlight the importance and raise sensibility on the necessity of a multidisciplinary approach; and (3) to provide clinicians with a practical tool aimed at improving the prompt recognition and follow-up of ILD associated with autoimmune rheumatic diseases (ARDs) and of any possible rheumatic conditions underlying ILD.

## Methods

**Figure 1:**
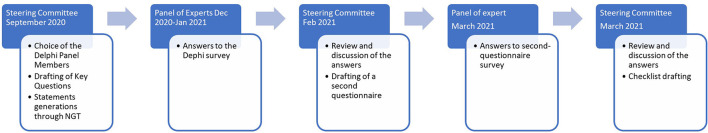
Project flowchart.

The project structure is shown in the flowchart (Figure 1).

Briefly, a Steering Committee reviewed the available literature and identified six key questions, which were used to generate some statements through the Nominal Group Technique (NGT) (16). The statements were used for a round of an adapted Delphi survey for an expert panel. Answers were used to draft a first checklist and as inputs to design a second, more specific questionnaire. Eventually, results from the second questionnaire were integrated with the result of the Delphi survey by the Steering Committee to define the check lists of red flags and the timing of ILD screening and monitoring.

### Steering Committee and Delphi Expert Panel

The members of both the Steering Committee and Delphi Panel are experts on ARD-associated ILDs.

The Steering Committee included different healthcare professionals, namely three pneumologists (SH, AP, FV), three rheumatologists (SB, NDP, MS), one immunologist (LB), and two radiologists (SP, GR). The Steering Committee sought the assistance of a non-clinical chair from an independent scientific consultancy agency (Polistudium srl, Milan, Italy) in order to provide meeting facilitation, material preparation and scientific accuracy. The Steering Committee designed and developed the project, identified the expert panel, generated the statements, reviewed and discussed survey results, and drafted the checklists.

The expert panel comprised members of different therapeutic areas in order to achieve a multidisciplinary overview. Inclusion criteria were clinical experience in ARD associated-ILDs and proven activity in MDTs. Candidate experts were proposed, shared, and approved within the Steering Committee.

### Literature Review and Key Questions

The Steering Committee with the help of the non-clinical chair reviewed the most recent literature on the topic and drafted six key questions to be used to generate statements through a NGT round. Domains of the questions comprised (1) risk factors; (2) pulmonary signs and symptoms; (3) rheumatological signs and symptoms; (4) monitoring timing and frequency of pulmonary symptoms in ARD and ARD-ILD patients; (5) rheumatologists' and pulmonologists' sensitivity and attention to the suspicion of ILD; and (6) how to implement multidisciplinary management.

### Statement Definition

The NGT is a direct and structured technique, based on experts' opinion, aimed at managing meetings organized to make decisions on a specific topic on which there is no strong evidence (16). The NGT was used to generate the statements for the Delphi Panel. At first, the six key questions were asked to the members of the steering committee, who then had the opportunity to independently develop their own thoughts and opinions during the “silent generation” process. Their opinions were presented during an online meeting in September 2020, chaired by a Professional Facilitator. All the opinions (items) were collected and shared with the participants; with the help of the facilitator, items were re-elaborated and similar ones were merged according to a statistical clustering and participants' opinion to draft preliminary statements. Before reaching a final formulation, participants had the opportunity to review and/or comment all items. The so-drafted 57 statements were ranked through an online survey (due to COVID-19 pandemic) in terms of priority and relevance using a 1–5 scale during a second, remotely performed meeting. All 57 statements were considered relevant and kept, eventually drafting the complete list of items.

### Adapted Delphi Process

The Delphi Method is a standard method of consensus, used to evaluate in an interactive and anonymous way, through online surveys, the level of agreement (consensus quantification) using a Likert scale (1–5; 1 = total disagreement; 5 = total agreement) and to resolve differences of opinion (consensus development). It takes place through several phases or rounds of expression and evaluation of opinions of a group of appropriately selected experts (17). Consensus on agreement is reached when at least 75% of voters express a vote equal to 4 or 5, according to the indications of the Ministry of Health (18).

Between December 2020 and January 2021, panelists participated to the Delphi online survey and indicated their level of agreement with the statements generated through the NGT.

### Second Questionnaire

A qualitative second online survey aimed at outlining, more precisely, what the red flags are and the timing of ILD screening and monitoring was drafted by the Steering Committee after the first Delphi round. The members of the Delphi Panel participated in the online survey.

### Statistical Analysis

All data were analyzed with descriptive statistics.

## Results

### NGT and Delphi Survey

During the NGT round, the Steering Committee answered six key questions and defined 57 statements to be used in the Delphi survey. The key questions statements are reported in Tables 1, 2.

**Table 1:** Statements that reached overall consensus.

**Statements that reached consensus**	**Level of agreement (%)**			
	**Total**	**Pn**	**Rh**	**Ra**
**Q1: What are the main risk factors for the development of ILD in ARDs?**				
1.1—ARDs, in particular, systemic sclerosis, rheumatoid arthritis, and anti-synthetase syndrome, have to be considered important risk factors for the development of ILD.	98	100	94	100
1.2—In the presence of ARDs, the presence of some autoantibodies (anti-JO1, anti-PL 7, anti-PL12, anti-SSA Ro, anti-MDA 5, anti-Scl70, anti-PM/Scl, anti-Th/To) increase the risk of developing ILD.	94	90	97	94
1.3—Some gene variants are associated with a greater risk of developing ILD, particularly for some forms, such as usual interstitial pneumonia.	78	87	**67**	84
1.4—In the case of a patient suffering from systemic sclerosis, there are specific risk factors for the development of ILD, such as male gender, a diffuse form of the disease, and the presence of anti-Scl70 antibodies.	87	81	97	78
1.6—In patients with rheumatoid arthritis, the risk of developing ILD increases in males, smoking patients, with an older age of onset, in proportion to the duration of disease, and the titer of anti-citrulline antibodies.	83	84	88	**72**
**Q2: What are the pulmonary signs, symptoms, and investigations that rheumatologists need to evaluate in generating a suspicion of ILD in patients with ARD?**				
2.1—A careful anamnesis about the presence of respiratory symptoms is essential in the ARD work-up to evaluate any symptoms of ILD.	91	97	85	94
2.2—A careful thoracic physical examination is essential in the work up of systemic autoimmune diseases to assess the presence of ILD.	87	97	76	94
2.3—The presence of dry cough and exertional dyspnea, not justified by an infectious respiratory or cardiological pathology in progress, can generate the suspicion of ILD in a patient with ARD.	97	100	94	100
2.4—The presence of a feeling of fatigue or chest tightness or digital hippocratism or chest pain can raise the suspicion of ILD in a patient with ARD.	84	86	82	83
2.5—Presence of basal velcro crackles on chest auscultation may raise the suspicion of ILD in a patient with ARD.	92	100	88	89
2.6—Chest x-ray is a poorly specific and insensitive tool to check for the presence of ILD in a patient with ARD.	86	76	97	83
2.7—Spirometry coupled with the CO diffusion test is an investigation to be performed to monitor the course of ILD in a patient with ARD.	97	97	97	100
2.8—The high-resolution CT scan of the chest is the most sensitive and specific radiological method to validate the presence of ILD in a patient with ARD.	100	100	100	100
2.9—If ILD is suspected, a volumetric rather than axial CT scan should be performed, with multiplanar reconstructions and eventual scans in prone decubitus.	89	90	82	100
2.10—Blood–gas analysis allows to evaluate the degree of impairment of gas exchange at rest during ILD in a patient with ARD.	80	79	79	83
2.11−6MWT (Six-Minute Walking Test) allows to evaluate the functional consequences of cardio-pulmonary damage during ILD in a patient with ARD.	80	90	**67**	89
2.12—In case of systemic sclerosis and anti-synthetase syndrome, high-resolution CT of the chest is already recommended at diagnosis.	85	83	88	83
2.13—Within the framing work-up of a diffuse type of systemic sclerosis in early phase or in the presence of predisposing antibodies, high-resolution chest CT scan must always be considered.	92	93	100	78
**Q3: What are the rheumatological signs and symptoms that pulmonologists need to evaluate in generating a suspicion of ARD in patients with ILD?**				
3.1—Presence of Raynaud's phenomenon, digital edema, skin sclerosis, digital ulcers, telangiectasias, alone or in combination, can generate the suspicion of ARD in a patient with ILD.	91	90	94	88
3.2—Presence of skin manifestations (lower limbs purpura, Gottron's papules, vasculitis, photosensitivity, palmar erythema) can lead to suspicion of ARD in a patient with ILD.	87	83	91	88
3.3—Presence of skin cracks on fingers (“mechanic's hands”) can lead to suspicion of ARD in a patient with ILD.	91	86	94	94
3.4—Presence of Sicca syndrome can raise suspicion of ARD in an ILD patient.	76	86	**73**	94
3.5—The presence of arthralgia and morning stiffness can generate suspicion of ARD in a patient with ILD.	77	80	76	77
3.7—Positivity to antinuclear antibodies of a significant titer or ≥1/320 may raise suspicion of ARD in a patient with ILD.	85	79	85	94
3.12—In patients with ILD, capillaroscopy should be required at least for patients with Raynaud's phenomenon and for those with specific autoantibodies for systemic sclerosis, mixed connective tissue disease and myositis (anticentromere, anti-Scl70, anti-RNP, specific anti-myositis, anti-synthetase)	100	100	100	100
**Q4: What should be the monitoring timing and frequency of pulmonary symptoms in the patient with ARD?**				
4.1—Pulmonary symptoms in the ARD patient should be assessed at each visit.	92	93	93	100
4.3—Timing and frequency of pulmonary symptoms monitoring in the ARD patient depend on the specific rheumatic disease.	78	76	82	77
4.4—In the event of worsening respiratory symptoms in a patient with ARD, a high-resolution chest CT scan should be performed.	87	100	76	88
4.5—Respiratory function tests and carbon monoxide alveolar–capillary diffusion test (DLCO) should be performed every 12 months in patients with ARD, in case of systemic sclerosis every 6–12 months.	86	90	82	88
4.6—Pulmonary symptoms of patients with systemic sclerosis should be monitored every 6 months in case of progressive rheumatic disease.	81	90	**72**	88
4.7—Respiratory function tests and carbon monoxide alveolar–capillary diffusion test (DlCO) should be performed every 12 months in the presence of clinical (systemic sclerosis) or laboratory (predisposing autoantibodies) risk factors in the absence of proven ILD.	90	93	88	88
**Q4.1: What should be the monitoring timing and frequency of pulmonary symptoms in the patient with ARD–ILD?**				
4.1.1—In case of patients with ARD and ILD, it is necessary, depending on the severity, to evaluate pulmonary symptoms every 3–6 months, carry out spirometry tests every 3–6 months, carry out carbon monoxide alveolar–capillary diffusion tests (DLCO), perform Six-Minute Walking Test (6MWT), and perform echocardiogram every 6–12 months.	85	90	90	**71**
4.1.2—Possible appearance or progression of ILD must be evaluated, in relation to the disease, by high-resolution CT scan of the chest as symptoms vary, in the presence of velcro crackles or worsening of functional tests.	98	97	97	100
**Q5: What could be the approaches to increase rheumatologists' and pulmonologists' sensitivity and attention to the suspicion of ILD, in the Italian setting?**				
5.1—The creation of a network between different centers of reference, which also favors the organization of national collaborative studies between pulmonologists, radiologists, and rheumatologists, can help to increase rheumatologists' and pulmonologists' sensitivity and attention to suspicion of ILD, in the Italian setting.	96	93	97	100
5.2—Webinar organization, seminars with MDT, and monothematic courses at regional and national level can be useful in increasing rheumatologists' and pulmonologists' sensitivity and attention to the suspicion of ILD, in the Italian setting.	87	90	79	100
5.3—Opportunity increase for meeting and updating with experts, through regional periodic scientific tables with simulation of MDT on paradigmatic cases or participation in multidisciplinary clinics, can be useful to increase rheumatologists' and pulmonologists' sensitivity and attention to the suspicion of ILD, in the Italian setting.	96	93	97	100
5.4—Sharing literature (e.g., creation of a six-monthly scientific bulletin to be distributed to level 1 centers) can be useful in increasing rheumatologists' and pulmonologists' sensitivity and attention to the suspicion of ILD, in the Italian setting.	77	79	**70**	88
5.5—The creation of an informatic platform, where level 1 centers can ask more specialized centers' opinion, can serve to increase rheumatologists' and pulmonologists' sensitivity and attention to the suspicion of ILD, in the Italian setting.	82	90	**73**	88
**Q6: What can be ways to implement multidisciplinary management of ARD patients with suspicion of ILD?**				
6.1—Creation of shared clinics between rheumatologists and pulmonologists can facilitate multidisciplinary management of rheumatology patients with suspicion of ILD.	95	93	100	82
6.2—Organization of joint training courses between rheumatologists, pulmonologists, radiologists can be useful for implementing multidisciplinary management of rheumatological patients with suspicion of ILD.	97	96	97	100
6.3—Creation of a preferential path of access to rheumatologists and pulmonologists for patients with suspected ILD, secondary to ARD, can favor multidisciplinary management of rheumatological patients with suspicion of ILD.	94	89	94	100
6.4—Sharing of diagnostic classification criteria of both ARD and ILD among the rheumatological and pneumological community, e.g., with the formulation of statements by scientific societies or the organization of regular meetings for the discussion of cases, would favor a multidisciplinary management of rheumatologic patients with suspicion of ILD.	95	100	88	100
6.5—In the case of patients with lung disease not classified with certainty by the pulmonologist, a rheumatological evaluation should also be performed.	82	75	82	94

*Pn, pneumologists; Rh, rheumatologists; Ra, radiologists. Values in bold highlight an unmet consensus within a specialty*.

In total, 85 experts from different therapeutic areas were invited to join the Delphi Panel (Supplementary Appendix A); all the members were based in Italy.

A total of 78 total experts participated in the Delphi survey, composed of 28 pulmonologists, 33 rheumatologists, and 17 radiologists. During this round, consensus on agreement was reached in 47 statements, as shown in Table 1. Consensus on disagreement was not reached in any statements. Statements in which consensus was not reached (*n* = 10) are shown in Table 2. Due to the practical aim of this paper (i.e., the creation of a checklist regarding useful red flags to suspect ILD in ARD patients and vice versa and regarding screening and monitoring of ILD in ARD patients) this process was adapted by doing a single round; the first-round responses were analyzed by the Steering Committee, who reviewed statements in which consensus had not been reached, discussed the reasons, and provided inputs for the creation of the second, more-in-depth questionnaire.

**Table 2:** Statements without overall consensus.

**Statements that reached consensus**	**Level of agreement (%)**			
	**Total**	**Pn**	**Rh**	**Ra**
**Q1: What are the main risk factors for the development of ILD in ARDs?**				
1.5—The severity of skin involvement in case of systemic sclerosis correlates with an increased risk of ILD.	51	26	**79**	44
1.7—The risk of developing ILD tends to increase with the age of onset of ARD, such as in the case of rheumatoid arthritis.	73	65	**79**	**78**
**Q3: What are the rheumatological signs and symptoms that pulmonologists need to evaluate in generating a suspicion of ARD in patients with ILD?**				
3.6—Presence of joint deformations can raise the suspicion of ARD in a patient with ILD.	71	**76**	61	**82**
3.8—Presence of alteration in phlogosis indexes can generate suspicion of ARD in a patient with ILD.	35	31	30	53
3.9—Morning functional impotence can raise suspicion of ARD in a patient with ILD.	53	55	49	59
3.10—Presence of subcutaneous nodules may raise suspicion of ARD in a patient with ILD.	66	62	58	**88**
3.11—Presence of a feeling of hyposthenia can generate suspicion of ARD in an ILD patient.	44	41	46	47
**Q4: What should be the monitoring timing and frequency of pulmonary symptoms in the patient with ARD?**				
4.2—Pulmonary symptoms in ARD patients should be monitored every 12 months for stable rheumatic disease or low-risk patients.	70	66	67	**82**
4.8—In the case of high-risk patients (i.e., diffuse systemic sclerosis with the presence of anti-scl70 antibodies) pulmonary symptoms should be evaluated every 3 months while high-resolution chest CT should be performed every 12 months.	72	**76**	67	**77**
**Q6: What can be ways to implement multidisciplinary management of ARD patients with suspicion of ILD?**				
6.6—Creation of “smart” digital platforms for each MDT group can facilitate multidisciplinary management of rheumatology patients with suspicion of ILD.	72	68	64	**94**

*Pn, pneumologists; Rh, rheumatologists; Ra, radiologists; Values in bold highlight a reached consensus within a specialty*.

### Second Questionnaire

This first round led to some reflections and conclusions from the committee: (1) there were clear difficulties for reaching consensus in some cases (cf. statement 1.5); differences in views holds despite all members being expert on the topic and part of MDTs—but this is where, in our opinion, value of multidisciplinary resides; (2) some answers from the Delphi survey were conflicting and weren't suitable with our need to give clear indications on patient management; (3) some statements were less relevant for the aim of the paper and were decided not to be investigated with a second round, while others were vague and interpretable. A further reason resides on the practical aim of this paper, which lead us in asking more specific questions to give clearer indications based on opinions of the large number of experts of the Delphi Panel. A detailed questionnaire, with a different structure than the Delphi, was thus drafted. This included four sections. In section A, which was addressed only to rheumatologists, the signs and symptoms they would report as red flags to pulmonologists to help suspect ILD in patients with ARD were ranked. Section B had the same structure but was only addressed to pulmonologists and which red flags they would report to the rheumatologist. Section C included questions regarding the tests—and their timing—to be performed on ARD patients without a diagnosis of ILD both in the presence and absence of risk factors for developing ILD. Section D questions were the same as section C but focused on ARD patients with a diagnosis of ILD and on risk factors for ILD progression rather than ILD developing. Questions in section C and D also asked to make the considered risk factors explicit, and to express any adjunctive comments; moreover, they did not only refer to a generic ARD patient, but specifically addressed the following rheumatic diseases: SSc, antisynthetase syndrome, Sjögren's syndrome, RA, and undifferentiated CTD.

A total of 76 clinicians (31 pulmonologists, 30 rheumatologists, and 15 radiologists) took part in the second survey. All the questions are available as Supplementary Material (Supplementary Tables).

### Red Flags of ILD in Patients With ARD

Rheumatologists should pose particular attention to signs and symptoms shown in Table 3 since they are useful red flags to suspect an underlying ILD in patients with ARD. If any of these is present, a high-resolution computed tomography (HRCT) should be prescribed.

**Table 3:** Check list of red flags sign or symptom suggestive of ILD.

Presence of basal velcro crackles on chest auscultation.
Dry cough and exertional dyspnea, not justified by an infectious respiratory or cardiological pathology in progress.

### Red Flags of ARD in Patients With ILD

Pulmonologists should pose particular attention to signs and symptoms shown in Table 4 since they are useful red flags to suspect an underlying ARD in patients with ILD. If any of these is present, patients should be referred to a rheumatologist.

**Table 4:** Check list of red flags sign or symptom suggestive of ARD.

Skin manifestations (cutaneous sclerosis, purpura of the lower limbs, Gottron's papules, cutaneous vasculitis, photosensitivity, palmar erythema, “mechanic's hands”).
Raynaud's phenomenon.
Digital ulcers and telangiectasias, alone or in combination.
Positivity to anti-nuclear antibodies with significant titer (≥1/160).
Presence of muscle weakness associated with an increase in CPK.
Arthralgia, joint swelling or swelling of the hands, morning stiffness.
Dry eyes and dry mouth.

### Screening and Monitoring of ILD in Patients With ARD

Following indications given from the expert panel through the Delphi survey and the second questionnaire, pulmonary symptoms in the ARD patient should be assessed at each visit (item 4.1, Table 1). Considering that the ARD-intrinsic risk of onset and developing of ILDs changes according to specific ARDs (items 1, Table 1) (19), the timing and type of screening and monitoring must be evaluated according to the specific pathology, and the overall clinical condition of the patient.

### ILD in Patients With RA

Clinically evident ILD is usually reported in 7–10% of patients with RA (20, 21), and lifetime risk of RA-ILD of 7.7% has been reported in a population-based cohort study conducted in the USA (22). However, prevalence largely varied according to the different studies and it is significantly higher when consecutive patients are evaluated by HRCT, recording abnormalities compatible with ILD in up to one-third of cases (23–25).

Although the few available data, generally based on retrospective studies, male sex, older age at RA onset, and ever-smokers are associated with RA-ILD in majority of studies (26, 27), mainly for patients with a usual interstitial pneumonia pattern.

Despite contrasting results, anti-citrullinated peptide antibodies (ACPA) have been also associated to ILD. In particular, Correia reported a correlation between ACPA titer and the risk to develop ILD (28). Finally, Doyle reported that a combination of older age, male sex, ever-smoking, RF, and ACPA was strongly associated with RA-ILD (29).

The Steering committee analyzed the answers from both the Delphi and the second survey, discussed such answers, compared them with available literature, integrated them with its opinion, and drafted a practical checklist for screening and monitoring of ILD in patients with RA, as shown in Table 5.

**Table 5:** Identification and monitoring of RA-ILD.

	Respiratory signs and symptoms^*^	**Spirometry and DLCO**	**HRCT**
Baseline/diagnosis time	Check	In presence of respiratory signs or symptoms^*^	In presence of respiratory signs or symptoms^*^
Follow-up in patients without a known ILD	Check at every examination^*^	In presence of respiratory signs or symptoms^*^ or when a pulmonary arterial hypertension is suspected^a^^,^^b^	In presence of respiratory signs or symptoms^*^ and/or in presence of significant deficit of functional tests^§^
Follow-up in patients with a known ILD	Check at every examination NB: Worsening of symptoms are suggestive of ILD progression or complications°	Every 3–6 months according to clinical status	Every 12 months according to clinical status^c^

a *Do not delay spirometry if DLCO is not available in a short time*.

b *Discrepancy between FVC and DLCO deficiency may suggest the presence of pulmonary hypertension*.

c *HRCT should be performed (1) in case of a worsening of clinical symptoms or lung function tests or (2) in stable patients to exclude lung cancer and to monitor lung disease*.

* *Presence of basal Velcro crackles, dry cough, and exertional dyspnea, not justified by a respiratory infection or cardiological pathology in progress*.

§ *FVC and/or TLC and/or DLCO deficit ≥20%*.

° *Infection, cancer, heart failure, drug toxicity*.

### ILD in Patients With SSc

Interstitial lung disease is a common manifestation of SSc, with approximately one-third of patients developing progressive ILD (30). Fibrotic non-specific interstitial pneumonia is the most common feature of parenchymal lung disease in patients with SSc-associated ILD, followed by usual interstitial pneumonia. Both the forms appear to have a similar survival in patients with SSc (31, 32). Despite significant improvement in the overall 10-year survival in SSc patients in the last few years, ILD represents a significant cause of morbidity and mortality. Risk factors for the development or progression of ILD among patients with SSc include diffuse cutaneous SSc, male sex, African–American race, older age at disease onset, shorter disease duration, and the presence of anti-Scl-70/anti-topoisomerase I antibody (33–36). However, none of these risk factors is absolute. Clinicians should remember that ILD may develop even in patients with limited cutaneous SSc. In addition, SSc-ILD has a variable and not predictable clinical course. Most patients experience a slow decline in lung function, but others have a rapid progression just after disease onset (37). Different studies showed that the most important predictors of mortality in patients with SSc-ILD are the short-term changes in pulmonary functional parameters (38, 39) and extent of lung fibrosis on HRTC. Despite physicians knowing the established relationship between SSc-ILD and mortality and morbidity well, the lack of a consensus on ILD screening, and monitoring of disease progression raise important implications for a better therapeutic management of SSc-ILD patients, mainly for new available treatment options.

The Steering committee analyzed the answers from both the Delphi and the second survey, discussed such answers, compared them with available literature, integrated them with its opinion, and drafted a practical checklist for screening and monitoring of ILD in patients with RA, as shown in Table 6.

**Table 6:** Identification and monitoring of SSc-ILD.

	Signs and symptoms^*^	**Spirometry and DLCO**	**HRCT**
Baseline/diagnosis time	Check	Yes	Yes
Follow-up in patients without a known ILD	Check at every examination	Every 6–12 months or in case of onset of respiratory signs or symptoms^∧^	Every 24 months Every 12 months in presence of risk factors^a^
Follow-up in patients with a known ILD	Check at every examination NB: Worsening of symptoms are suggestive of ILD progression or complications°	Every 6–12 months, or every 3–6 months, if risk factors^a^ are present	To be performed every 12 months according to clinical status In case of rapid deterioration, re-evaluate the timing

a *Risk factors should be assessed at every examination. Risk factors include male gender, diffuse skin disease, and presence of anti-Scl70 antibodies*.

* *Presence of basal velcro crackles, dry cough and exertional dyspnea, not justified by a respiratory infection or cardiological pathology in progress*.

° *Infection, cancer, heart failure, drug toxicity*.

∧ *Do not delay spirometry if DLCO is not available in a short time*.

### ILD in Patients With Other ARD

The other ARD considered in our questions are systemic lupus erythematosus, Sjögren's syndrome, mixed CTD, polymyositis/dermatomyositis, undifferentiated CTD. The risk to develop an ILD varies between different diseases, but in some the mortality related to interstitial lung involvement is very high (i.e., in the antisynthetase syndrome, 28% developed progressive respiratory failure and died) (40). The high heterogeneity spectrum of these diseases either in the risk to develop an ILD either in clinical manifestations and in progression of lung involvement reduced the consensus of the statement that varies from 5 to 75% in the timing of performing CT scan and from 4.5 to 70% in timing to a perform function test.

Regarding identification and monitoring of ILD associated with these ARDs, the respiratory signs and symptoms to be valued are the same as the ones presented for RA and SSc. Diffusing capacity of the lungs for carbon monoxide (DLCO) should be performed annually, or every 3–6 months in case of an already diagnosed ILD. In patients without an ILD diagnosis, HRCT should be performed when clinically indicated from symptoms, or, in patients at high risk for the clinical characteristics of the disease, every 12–24 months. If ILD has been already diagnosed, HRCT should be carried out at least annually.

### The Multidisciplinary Approach

All statements addressing how to increase sensitivity and attention to the suspicion of ILD in the Italian setting (Q5 and Q6, Table 1) reached consensus. Their approaches include the creation of a network between different centers of reference, webinar/seminar with MDT, monothematic courses, regional periodic scientific tables with simulation of MDT on paradigmatic cases or participation in multidisciplinary clinics, sharing literature, and the creation of an informatic platform, where level 1 centers can ask more specialized centers' opinion.

Statements referring to key question number 6 addressed how to implement multidisciplinary management of ARD patients with suspicion of ILD. Statements that reached consensus suggest the creation of shared clinics between rheumatologists and pulmonologists, the organization of joint training courses between rheumatologists, pulmonologists, and radiologists, the creation of a preferential path of access to rheumatologists and pulmonologists for patients with suspected ILD secondary to ARD, and sharing of diagnostic classification criteria of both ARD and ILD among the rheumatological and pneumological community. The only statement (6.6, Table 2) that did not reach consensus suggested the creation of “smart” digital platforms for each MDT group. However, consensus for this statement was reached among the radiologists, likely because of them being more prone in working in a digital setting given their every-day work always involves computers.

## Discussion

Interstitial lung disease is often associated with rheumatic diseases. Its early diagnosis and management are not only difficult, but also crucial, because it is associated with major morbidity and mortality and can be the first cause of death in ARDs (7–13). We, therefore, aimed to measure consensus between specialists who can be involved in its management: this is one of the very first studies to address consensus between pulmonologists, rheumatologists, and radiologists. Consensus was high, with 42 out of 50 statements that reached the 75% threshold agreement. No statements reached the disagreement threshold. With this work we also aimed at highlighting the importance of a multidisciplinary approach that includes rheumatologists, and at providing the drafted checklists (see Tables 3–6) as a practical tool useful in the prompt recognition and in follow-up of ARD-ILD.

The main strength of this study is the combinations of techniques, such as NGT and Delphi Survey, which allow clinicians firstly to share their own opinion rising from their personal experience, and secondly to work toward an integration of such opinions. This methodology highlights the multidisciplinary approach of this work. The importance of multidisciplinary approaches has been consolidated in the clinical practice, and it is of utmost important to keep such an approach for diagnosis, therapy and follow-up. The evaluation of ILD by an MDT has been proposed as the gold standard for its management (41) but, while up to 20% of ILD cases can be referred to rheumatic conditions, only ~37% of MDT cases worldwide include a rheumatologist (42); this may create a vicious circle, where rheumatologist referral is up to pulmonologist, who may underestimate clinical manifestation of an ARD. Therefore, if we also consider that ILD can be the leading cause of death for some ARD, and the exclusion of any systemic ARD in any freshly diagnosed ILD is mandatory according to current guidelines, we believe that rheumatologists' non-inclusion in MDTs is not justifiable; their view could potentially complete the evaluation of a pulmonologist, who may overlook important details (15). With regards to this study, when comparing factors taken into account by pneumologists and rheumatologists to decide on the monitoring of the exams, it shows such factors are more lung-related for pneumologists, and more disease-specific for rheumatologists. Clinicians should be aware of this “bias” since it could lead them in taking a wider perspective on the pathology in exam.

Despite the high reached consensus, when we take a more in-depth look to data from the surveys, and consider discussions of the meetings of the steering committee, some discrepancies arose in terms of attitude and management methods of the disease among Delphi panelists, the steering committee, and among clinicians of different expertise. For example, statement 1.5 “The severity of skin involvement in the case of SSc correlates with an increased risk of ILD” reached a level of agreement of 77.8% within rheumatologists, 44.4% between radiologists and 25.8% within pneumologists. While it may be that disagreement occurred because of actual lack of general knowledge or evidence, it could also be argued that agreement occurred for the same reasons. However, we believe this is not the case, since members of the expert panel were chosen for their clinical experience in ARD associated-ILDs and proven activity in MDTs. We think result heterogenicity from the Delphi survey can be explained in several different ways: the presence of specialists with different backgrounds and sensitivities, and with specific experience on different rheumatic diseases; the heterogeneity of rheumatic diseases themselves, which require approaches that cannot be generalized tout-court; a lack of international guidelines (except, partially, on idiopathic pulmonary fibrosis), which may have led panelists in sharing what they can do to the best of the means at their disposal in the everyday clinical setting; the need to reconcile the evaluation of pulmonary involvement with that of the other systemic manifestations of the disease; the difficulties of working in an MDT. Despite discrepancies arising from the variability of different points of view, we managed to integrate such diverse opinions through several meetings in which statements were discussed, compared to available literature, and our clinical view, and we went so far as to give our opinion based on our experience in MDTs. Notwithstanding differences between specialists, some statements reach a really strong consensus, with one of those being number 2.8 stating that “the high-resolution CT scan of the chest is the most sensitive and specific radiological method to validate the presence of ILD in a patient with ARD,” and reaching a level of agreement of 100%. This is coherent with HRCT driving therapeutic choices, given the fact that is useful to identify subclinical outlines, and differentiate ILA from subclinical forms of ARD-ILD (19).

Training clinicians and improving their sensitivity and attention to the suspicion of ARD-ILD can be a valuable solution when working with an MDT is not possible; this happens quite often in the Italian scenario, where the triplet pulmonologist, rheumatologist, and radiologist is not always available, or present within the same structure. Q5 of the Delphi survey addressed how to implement such training; results therefore show which ways clinicians would feel effective if they had to be instructed. The two most-agreed ways are the creation of a network between different centers of reference, which also favors the organization of national collaborative studies between pulmonologists, radiologists, and rheumatologists, and an opportunity increase for meeting and updating with experts, through regional periodic scientific tables with simulation of MDT on paradigmatic cases or participation in multidisciplinary clinics. Other solutions include webinars/seminars with MDT and monothematic courses at regional and national level, the creation of an informatic platform, where level 1 centers can ask more specialized centers' opinion, and sharing literature through scientific bulletin to be distributed to level 1 centers. Improving untrained physicians' sensitivity is the first step toward implementation of ARD-ILD multidisciplinary management. Q6 of the Delphi survey addressed ways for such implementations; statement consensus was reached for four out of five statements. According to the Delphi panelists, the most effective way to implement multidisciplinary management is the organization of joint training courses between rheumatologists, pulmonologists, and radiologists, followed by the creation of shared clinics between rheumatologists and pulmonologists, the sharing of diagnostic classification criteria of both ARD and ILD among the rheumatological and pneumological community (e.g., the formulation of statements by scientific societies or the organization of regular meetings for the discussion of cases), and eventually the creation of a preferential path of access to rheumatologists and pulmonologists for patients with suspected ILD secondary to ARD. On top of this, it must be remembered that being part of an MDT is an ongoing process. Even once multidisciplinary management has been implemented, clinicians need time to adapt to it: levels of agreement between different specialists rise over time, improving diagnostic, and managing performance (15, 43).

The solutions proposed in the statements from Q5 and Q6 could be effective, but they require a lot of time to be carried on and applied. Moreover, not all clinical settings are suitable for having an MDT. Because of this, and to provide all specialties with tools that are shared and recommended by other specialists. We propose some checklists to help recognition and follow up of ARD-ILD; these checklists arise from the integration of results from the Delphi survey, the second questionnaire, and our experience of MDT. Given the irreversibility and high morbidity and mortality rates of ILD (1, 44) a prompt diagnosis is extremely important; the red-flag checklist of respiratory signs and symptoms suggestive of ILD in ARD patients (Table 3) can be a useful tool for rheumatologists for the recognition of ILD. On the other hand, the red-flag checklist of systemic signs and symptoms suggestive of ARD in ILD patients (Table 4) is addressed to pulmonologist to help them recognize a rheumatic condition underlying ILD. A fast recognition of the presence of an ARD underlying ILD, and vice versa, can help guide therapy and give better outcomes. Tables 5, 6 go more in-depth tackling identification and monitoring of ILD in RA and SSc, respectively. They explicit symptoms to be addressed and examinations to be performed and give indication on the timing depending on whether the rheumatic disease has just been diagnosed, or patients are in the follow-up phase with a diagnosed or undiagnosed ILD already. We believe these short, easy-to-consult checklists can help untrained physicians better address these pathologies in the wait of more robust, international guidelines.

This study has four main limitations. Firstly, drafting items and statement can often lead to them being redundant or already addressed in literature Secondly, Delphi panelists could not comment on the relevance or importance of the drafted statements, as well as they could not give a position of “non-opinion.” Thirdly, it is limited to the Italian scenario; this may have yielded results that are not in line with other countries' reality, especially when considering every-day clinical practice, which can differ because of different regulations and resources. Finally, albeit based on the experience of a high number of clinicians, it is a consensus work and could not produce empiric data.

## Conclusions

This consensus work showed a high level of agreement, but also shows some divergent opinions between different experts. This underlines the importance of a multidisciplinary approach and of a constant update in overcoming these differences and in enhancing the diagnosis timing and management of patients with ILD-ARD. Given the high morbidity and mortality rates of ILD-ARD, its early recognition is crucial. The expert-shared red-flag checklist of respiratory signs and symptoms suggestive of ILD in ARD patients (Table 3) can be a useful tool for rheumatologists for the recognition of ILD, while the expert-shared red-flag checklist of systemic signs and symptoms suggestive of ARD in ILD patients can help the pulmonologist to recognize a rheumatic condition underlying ILD (Table 4). Since RA and SSc are two of the most common ARDs that can be associated with ILD, we drafted related checklists on identification and monitoring (Tables 5, 6), which can help tackle these conditions.

## Data Availability Statement

The raw data supporting the conclusions of this article will be made available by the authors, without undue reservation.

## Author Contributions

All authors contributed equally to the planning, implementation, and drafting of the study.

## Funding

The study was funded by an unrestricted grant from Boehringer Ingelheim Italia S.p.A.

## Author Disclaimer

BI was given the opportunity to review the manuscript for medical and scientific accuracy, as well as intellectual property considerations.

## Conflict of Interest

LB reports personal fees from Boehringer Ingelheim, Janssen, GSK, AZ, Werfen. SH has relationships with drug companies Boehringer Ingelheim and Roche. In addition to being investigator in trials involving these companies, relationships include lectures and membership of scientific advisory boards and grant for research. FV reports personal fees from Boehringer Ingelheim and Roche. MS reports personal fees from Boehringer Ingelheim, Janssen, Pfizer, Lilly, Bristol-Myers Squibb. SB has relationships with drug companies Boehringer Ingelheim, Roche, Janssen, AbbVie, and Pfizer. Relationships include lectures and membership of scientific advisory boards. SP reports personal fees from Boehringer Ingelheim and Roche SpA, not related to this submitted paper. GR reports personal fees from Boehringer Ingelheim and Roche SpA, not related to this submitted paper. AP has relationships with drug companies Boehringer Ingelheim and Roche. In addition to being investigator in trials involving these companies, relationships include lectures and membership of scientific advisory boards and grant for research. ND has relationships with drug companies Boehringer Ingelheim and Jansenn, Relationships include lectures and membership of scientific advisory boards. This study received funding from Boehringer Ingelheim. The funder was not involved in the study design, collection, analysis, interpretation of data, the writing of this article or the decision to submit it for publication.

## Publisher's Note

All claims expressed in this article are solely those of the authors and do not necessarily represent those of their affiliated organizations, or those of the publisher, the editors and the reviewers. Any product that may be evaluated in this article, or claim that may be made by its manufacturer, is not guaranteed or endorsed by the publisher.
