# Supplementary material for: Interstitial Lung Disease Associated With Autoimmune Rheumatic Diseases: Checklists for Clinical Practice
Source: Front Med (Lausanne). 2021 Oct 15;8:732761. doi: 10.3389/fmed.2021.732761 (PMC8554062; doi:10.3389/fmed.2021.732761)
Supplement: Supplementary file 1 [file Data_Sheet_1.docx]

## Appendix A: Delphi Panel members

| Name | Last Name | Affiliation |
| --- | --- | --- |
| Elisa | Baratella | Università di Trieste, SOC di Radiologia Diagnostica ed Interventistica |
| Carlo | Vancheri | Università di Catania |
| Gaetano | Rea | Dipartimento dei Servizi Diagnostici e Generali Ospedale Monaldi AO dei Colli |
| Sebastiano | Torrisi | Università di Catania |
| Maria | De Santis | Rheumatology, Humanitas University |
| Paola | Cipriani | Università degli Studi dell'Aquila |
| Ada | Vancheri | Ospedale San Marco |
| Stefano | Marinari | UOSD Pneumologia Ospedale SS Annunziata |
| Alberto | Ricci | Sapienza |
| Patrizio | Viiulo | IRCCS ISMETT |
| Ermanno | Puxeddu | Università degli studi di Roma Tor Vergata |
| Paolo | Ceruti | ASST Spedali Civili di Brescia |
| Giuseppe | Cicchetti | Fondazione Policlinico "A. Gemelli" IRCCS |
| Giorgia | Dalpiaz | AUSL Citta Di Bologna |
| Antonina | Minniti | MD, UOC Reumatologia, ASST Gaetano Pini CTO |
| Serena | Guiducci | SODc Reumatologia, Dipartimento di Medicina Sperimentale e Clinica, Università di Firenze |
| Gian Luca | Erre | Università degli Studi di Sassari e AOU di Sassari |
| Sebastiano Emanuele | Torrisi | Università di Catania |
| Alberto | Ricci | Sapienza Università di Roma |
| Chiara | Romei | Azienda Ospedaliero Universitaria Pisana |
| Lucio | Calandriello | Fondazione Policlinico Universitario A. Gemelli |
| Alfredo | Sebastiani | Az Osp San Camillo-Forlanini |
| Paola | Franchi | AUSL Teramo |
| Andreina | Manfredi | UO Reumatologia, Azienda Ospedaliero Universitaria Policlinico di Modena |
| Antonella | Caminati | U.O. di Pneumologia, Ospedale San Giuseppe Multimedica |
| Veronica | Codullo | IRCCS policlinico San Matteo |
| Adele | Valentini | Fondazione IRCCS policlinico San Matteo |
| Alessandra | Della Rossa | Aoup |
| Giovanni | Della Casa | Azienda Ospedaliero-Universitaria di Modena - Policlinico |
| Simone | Barsotti | UO Reumatologia AOU Pisana |
| Gianluca | Moroncini | Clinica Medica, Università Politecnica delle Marche & Azienda Ospedali Riuniti Ancona |
| Andrea | Borghesi | Department of Medical and Surgical Specialties, Radiological Sciences and Public Health, University of Brescia |
| Gerlando | Natalello | UOC di Reumatologia, Fondazione Policlinico Universitario A. Gemelli IRCCS - Istituto di Reumatologia, Università Cattolica del Sacro Cuore |
| Chiara | Baldini | Uo Reumatologia |
| Ennio Vincenzo | Sassani | Policlinico Foggia |
| Giacomo | De Luca | IRCCS Ospedale San Raffaele |
| Bruno | Iovene | Fondazione Policlinico Universitario A. Gemelli IRCCS |
| Alberto | Pesci | Pneumologia, Università Milano Bicocca |
| Alessandra | Farchione | Fondazione Policlinico Universitario Agostino Gemelli IRCCS |
| Elisa | Baratella | Università degli Studi di Trieste, Dipartimento di Scienze Mediche Chirurgiche e della Salute, UCO di Radiologia |
| Valeria | Riccieri | Sapienza Università di Roma |
| Sara | Tomassetti | Department of Experimental and Clinical Medicine, Careggi University Hospital |
| Anna Rita | Larici | Università Cattolica del Sacro Cuore di Roma |
| Ilaria | Cavazzana | Uo Reumatologia, ASST Spedali Civili |
| Paolo | Airò | Spedali Civili |
| Gianluca | Sambataro | Regional Referral Center For Rare Lung Disease, "Policlinico-San Marco", University of Catania |
| Roberta | Priori | uoc reumatologia Policlinico Umberto I |
| Giuseppina | Bertorelli | Università di Parma |
| Valentina | Vespro | Fondazione IRCCS Ca' Granda Ospedale Maggiore Policlinico |
| Marta | Vadacca | Campus Bio Medico |
| Mario | Silva | Università di Parma |
| Corrado | Campochiaro | Unit of Immunology, Rheumatology, Allergy and Rare Diseases (UnIRAR) IRCCS San Raffaele Hospital, Vita-Salute San Raffaele University |
| Renato | Carignola | AOU San Luigi Gonzaga |
| Francesco | Bini | ASST-Rhodense |
| Paola | Tomietto | SS di Reumatologia c/o SOC MEDICINA CLINICA, Ospedale di Cattinara |
| Caterina | Conti | Pneumologia - ASST Papa Giovanni XXIII |
| Elena | Bargagli | Università di Siena, AOUS |
| Lorenzo | Cavagna | Università di Pavia |
| Alarico | Ariani | DH Reumatologia Azienda Ospedaliero Universitaria di Parma |
| Emanuela | Barisione | IRCCS Ospedale Policlinico San Martino |
| Martina | Bonifazi | Università Politecnica Delle Marche |
| Mario | Salio | Azienda Ospedaliera SS Antonio e Biagio e C. Arrigo |
| Michele | De Ceglie | Policlinico |
| Serena | Vettori | UOC di Malattie, Fisiopatologia e Riabilitazione Respiratoria, Ospedale Monaldi |
| Giancarlo | Cortese | Ospedale Maria Vittoria |
| Fabrizio | Luppi | Università degli Studi Milano Bicocca |
| Tiberio | Oggionni | IRCCS Policlinico San Matteo |
| Armando | Gabrielli | Dipartimento di Scienze Cliniche e Molecolari |
| Roberta | Polverosi | AFFIDEA - IDA |
| Stefania | Cerri | Centro per le Malattie Rare del Polmone - AOU Policlinico di Modena |
| Cosimo | Bruni | Università degli Studi di Firenze |
| Carlo | Vancheri | Università di Catania |
| Almerico | Marruchella | ASST Monza - Ospedale San Gerardo Clinica Pneumologica |
| Donato | Lacedonia | Università di Foggia |
| Elisabetta | Zanata | UOC Reumatologia, Universita' Degli Studi Di Padova |
| Fabio | Cacciapaglia | U.O.C. Reumatologia Universitaria - AOUC Policlinico di Bari. DETO Università degli Studi di Bari |
| Edoardo | Rosato | Sapienza Università di Roma-Dipartimento di Medicina Traslazionale e di Precisione |
| Claudia | Ravaglia | Pneumologia, Ospedale GB Morgagni |
| Antonella | Arcadu | ASST Crema |
| Alessandra | Vacca | S.C. di Reumatologia, Azienda Ospedaliera Universitaria di Cagliari |
| Michele | Mondoni | Clinica di Malattie Respiratorie dell'Università degli Studi di Milano |
| Alice | Biffi | Sant’Anna |
| Giulia | Cassone | UO Reumatologia, Azienda Ospedaliero Universitaria Policlinico di Modena |
| Enrico | De Lorenzis | Università Cattolica del Sacro Cuore/Università di Verona |
| Roberto | Cassandro | Ospedale san Giuseppe |

## Appendix B: Second questionnaire

### RED FLAGS of ILD in patients with ARD

Results regarding the ranking of pulmonary signs that rheumatologists should pose particular attention to (Section A) are shown in the table below

| **Section A – only pulmonologists answered** | Score (1-3) |
| --- | --- |
| **Which of these signs would you like to point out to the rheumatologist as RED FLAGS on which to pay particular attention in a patient with ARD, since they could lead to a suspicion of ILD?** | |
| Presence of basal Velcro crackles on chest auscultation | 2.70 |
| Dry cough and exertional dyspnoea, not justified by an infectious respiratory or cardiological pathology in progress | 2.13 |
| Feeling of fatigue or chest tightness or digital hippocratism or chest pain | 1.17 |

### RED FLAGS of ARD in patients with ILD

Results regarding the ranking of rheumatological signs that pneumologists should pose particular attention to (Section B) are shown in the table below

| **Section B – only rheumatologists answered** | Score (1-9) |
| --- | --- |
| **Which of these signs would you like to point out to the pulmonologist as RED FLAGS on which to pay particular attention in a patient with ILD, since they are useful in raising a suspicion of ARD?** | |
| Skin manifestations (cutaneous sclerosis, purpura of the lower limbs, Gottron's papules, vasculitis, photosensitivity, palmar erythema, "mechanic's hands") | 8 |
| Raynaud's phenomenon | 6.89 |
| Digital ulcers and telangiectasias, alone or in combination | 6.50 |
| Positivity to anti-nuclear antibodies with significant titre (≥1 / 320) | 5.61 |
| Presence of stenic deficiency associated with an increase in CPK | 5.36 |
| Arthralgia, joint swelling or swelling of the hands, morning stiffness | 4.79 |
| Dry eyes and dry mouth | 3.54 |
| Persistent alteration of phlogosis indices not otherwise justifiable | 2.46 |
| Presence of subcutaneous nodules / joint deformities | 1.86 |

### The following questions refer to patients with specific ARDs but no ILD

1. In a patient with **sclerodermia** but with no evidence of ILD
   1. How often would you perform a CT scan?

- I wouldn't perform a CT scan without symptoms
- 3 months
- 6 months
- 12 months
- 24 months
  1. How often would you perform respiratory function tests?
- I wouldn’t perform tests without symptoms
- 3 months
- 6 months
- 12 months
- 24 months

1. In a patient with **antisynthetase syndrome** but with no evidence of ILD
   1. How often would you perform a CT scan?

- I wouldn't perform a CT scan without symptoms
- 3 months
- 6 months
- 12 months
- 24 months
  1. How often would you perform respiratory function tests?
- I wouldn’t perform tests without symptoms
- 3 months
- 6 months
- 12 months
- 24 months

1. In a patient with **Sjögren syndrome** but with no evidence of ILD
   1. How often would you perform a CT scan?

- I wouldn't perform a CT scan without symptoms
- 3 months
- 6 months
- 12 months
- 24 months
  1. How often would you perform respiratory function tests?
- I wouldn’t perform tests without symptoms
- 3 months
- 6 months
- 12 months
- 24 months

1. In a patient with **rheumatoid arthritis** but with no evidence of ILD
   1. How often would you perform a CT scan?

- I wouldn't perform a CT scan without symptoms
- 3 months
- 6 months
- 12 months
- 24 months
  1. How often would you perform respiratory function tests?
- I wouldn’t perform tests without symptoms
- 3 months
- 6 months
- 12 months
- 24 months

1. In a patient with **other ARD(s)**, but with no evidence of ILD
   1. How often would you perform a CT scan?

- I wouldn't perform a CT scan without symptoms
- 3 months
- 6 months
- 12 months
- 24 months
  1. How often would you perform respiratory function tests?
- I wouldn’t perform tests without symptoms
- 3 months
- 6 months
- 12 months
- 24 months

### The following questions refer to patients being diagnosed with specific ARDs and ILD

1. At the time of diagnosis, in a patient with **sclerodermia and ILD**
   1. How often would you perform a CT scan?

- I wouldn't perform a CT scan without symptoms
- 3 months
- 6 months
- 12 months
- 24 months
  1. How often would you perform respiratory function tests?
- I wouldn’t perform tests without symptoms
- 3 months
- 6 months
- 12 months
- 24 months

1. At the time of diagnosis, in a patient with **antisynthetase syndrome and ILD**
   1. How often would you perform a CT scan?

- I wouldn't perform a CT scan without symptoms
- 3 months
- 6 months
- 12 months
- 24 months
  1. How often would you perform respiratory function tests?
- I wouldn’t perform tests without symptoms
- 3 months
- 6 months
- 12 months
- 24 months

1. At the time of diagnosis, in a patient with **Sjögren syndrome and ILD**
   1. How often would you perform a CT scan?

- I wouldn't perform a CT scan without symptoms
- 3 months
- 6 months
- 12 months
- 24 months
  1. How often would you perform respiratory function tests?
- I wouldn’t perform tests without symptoms
- 3 months
- 6 months
- 12 months
- 24 months

1. At the time of diagnosis, in a patient with **rheumatoid arthritis and ILD**
   1. How often would you perform a CT scan?

- I wouldn't perform a CT scan without symptoms
- 3 months
- 6 months
- 12 months
- 24 months
  1. How often would you perform respiratory function tests?
- I wouldn’t perform tests without symptoms
- 3 months
- 6 months
- 12 months
- 24 months

1. At the time of diagnosis, in a patient with **undifferentiated connective tissue disease and ILD**
   1. How often would you perform a CT scan?

- I wouldn't perform a CT scan without symptoms
- 3 months
- 6 months
- 12 months
- 24 months
  1. How often would you perform respiratory function tests?
- I wouldn’t perform tests without symptoms
- 3 months
- 6 months
- 12 months
- 24 months

1. At the time of diagnosis, in a patient with **other ARD(s) and ILD**
   1. How often would you perform a CT scan?

- I wouldn't perform a CT scan without symptoms
- 3 months
- 6 months
- 12 months
- 24 months
  1. How often would you perform respiratory function tests?
- I wouldn’t perform tests without symptoms
- 3 months
- 6 months
- 12 months
- 24 months

### The following questions refer to patients already diagnosed with specific ARDs and ILD and during follow-up

1. During follow up, in a patient with **sclerodermia and ILD**
   1. How often would you perform a CT scan?

- I wouldn't perform a CT scan without symptoms
- 3 months
- 6 months
- 12 months
- 24 months
  1. How often would you perform respiratory function tests?
- I wouldn’t perform tests without symptoms
- 3 months
- 6 months
- 12 months
- 24 months

1. During follow up, in a patient with **antisynthetase syndrome and ILD**
   1. How often would you perform a CT scan?

- I wouldn't perform a CT scan without symptoms
- 3 months
- 6 months
- 12 months
- 24 months
  1. How often would you perform respiratory function tests?
- I wouldn’t perform tests without symptoms
- 3 months
- 6 months
- 12 months
- 24 months

1. During follow up, in a patient with **Sjögren syndrome and ILD**
   1. How often would you perform a CT scan?

- I wouldn't perform a CT scan without symptoms
- 3 months
- 6 months
- 12 months
- 24 months
  1. How often would you perform respiratory function tests?
- I wouldn’t perform tests without symptoms
- 3 months
- 6 months
- 12 months
- 24 months

1. During follow up, in a patient with **rheumatoid arthritis and ILD**
   1. How often would you perform a CT scan?

- I wouldn't perform a CT scan without symptoms
- 3 months
- 6 months
- 12 months
- 24 months
  1. How often would you perform respiratory function tests?
- I wouldn’t perform tests without symptoms
- 3 months
- 6 months
- 12 months
- 24 months

1. During follow up, in a patient with **undifferentiated connective tissue disease and ILD**
   1. How often would you perform a CT scan?

- I wouldn't perform a CT scan without symptoms
- 3 months
- 6 months
- 12 months
- 24 months
  1. How often would you perform respiratory function tests?
- I wouldn’t perform tests without symptoms
- 3 months
- 6 months
- 12 months
- 24 months

1. During follow up, in a patient with **other ARD(s) and ILD**
   1. How often would you perform a CT scan?

- I wouldn't perform a CT scan without symptoms
- 3 months
- 6 months
- 12 months
- 24 months
  1. How often would you perform respiratory function tests?
- I wouldn’t perform tests without symptoms
- 3 months
- 6 months
- 12 months
- 24 months
